# Symptoms of anxiety and depression in surgical patients at the hospital, 6 weeks and 6 months postsurgery: A questionnaire study

**DOI:** 10.1002/nop2.620

**Published:** 2020-09-16

**Authors:** Herdís Sveinsdóttir, Sigríður Zoëga, Brynja Ingadóttir, Katrín Blöndal

**Affiliations:** ^1^ Faculty of Nursing University of Iceland Reykjavík Iceland; ^2^ Surgical Services Landspitali University Hospital Reykjavík Iceland

**Keywords:** anxiety, depression, nursing, perioperative care, surgical patients

## Abstract

**Aims:**

To describe prevalence of symptoms of anxiety and depression in surgical patients at three time points: at hospital postsurgery (T1), 6 weeks (T2) and 6 months (T3) postdischarge from hospital; and detect situations and experiences that predict symptoms of anxiety and depression at T2 and T3.

**Design:**

Prospective, explorative two‐site follow‐up study.

**Methods:**

Patients having selected surgeries from January–July 2016 were invited to participate. Final participation was 390 patients. Participation involved answering questionnaires, including the Hospital Anxiety and Depression Scale (HADS). A stepwise multiple linear regression model was employed to calculate predictors of anxiety and depression.

**Results:**

The proportion of patients presenting with moderate‐to‐severe anxiety or depression ranged from 5.4%–20.2% at different times. Major predictors of anxiety at both times were not feeling rested upon awakening and higher scores on HADS‐Anxiety at T1 and T2 and at T2 also experiencing more distressing postoperative symptoms. For depression, the major predictors were at both times higher scores on HADS‐Depression at T2 and T3 and also at T2 not feeling rested upon awakening and at T3 reporting delayed or very delayed recovery.

The four models explained from 43.9%–55.6% of the variance in symptoms of anxiety and depression. Our findings show that patients presenting with psychological distress at the hospital are in a vulnerable position. Also, that benefits of good sleep during the recovery should be emphasized during hospital stay.

**What does this paper contribute to the wider global clinical community?**

- Psychological distress of surgical patients is real, and those who organize and provide care for surgical patients should take this reality into consideration when organizing care.
- This research showed a relationship between delayed recovery, feeling unrested and experiencing distress as a result of postoperative symptoms with psychological distress up to 6 months postdischarge from hospital. Routine methods to detect those at risk for long‐term psychological distress should be implemented and those at risk followed for at least 6 months.
- Due to the health consequences of untreated depression/anxiety, those who meet criteria for having moderate‐to‐severe symptoms should be referred to appropriate health professionals for medical or psychological treatment.

## 1 INTRODUCTION

The long‐term aim of nursing care for surgical patients is to safely deliver a recovered patient back to society or his/her normal activities. This is accomplished through nursing care at the hospital, discharge education (Fredericks and Yau, 2013; Kang et al., 2020), postdischarge follow‐up and intervention if needed. Outcome measurements of surgery and care are often rehospitalization (Fischer et al. 2014), length of stay at the hospital (Lingsma et al., 2018), return to work/normal activities and resolution of various symptoms depending on the disease leading to surgery (Lee, Tran, Mayo, Carli, & Feldman, 2014). This can be influenced by the patient's health in general, family and social situation, age and gender (Lee et al., 2014). The focus of nursing care for surgical patients during the immediate postoperative period is mainly on providing safe physical care. However, in consequence of long‐term effects of symptoms of anxiety and depression, it is necessary to address these also (Basak et al., 2015; Geulayov, Novikov, Dankner, & Dankner, 2018; Yilmaz, Sezer, Gürler, & Bekar, 2012). There is ample evidence to suggest that the presence of pre‐operative depression is associated with postsurgical comorbidities and even mortality, particularly among coronary artery bypass surgery (CABG) patients (Geulayov et al., 2018; Stenman, Holzmann, & Sartipy, 2016). Likewise, unresolved pre‐operative anxiety can lead to unnecessary pain, more analgesic and anaesthetics use, longer hospital stays and infection as well as other complications (Carr, Thomas, & Wilson‐Barnet, 2005; Tully et al., 2015).

This study seeks to identify situations and experiences that are present during the recovery period and that may be associated with psychological disturbances manifested in symptoms of anxiety and depression up to 6 months following an elective surgical procedure. The presence of symptoms of these disturbances is not tantamount to depressive or anxiety disorder. However, identifying those situations and experiences during hospitalization or early recovery may prompt timely and appropriate detection of and intervention for these disturbances.

### 1.1 Background

Depression is ranked by the World Health Organization as the number one contributor to global disability (7.5% of all years lived with disability in 2015), and anxiety disorders are ranked in sixth place (3.4%) (WHO 2017). The consequences of these disorders in terms of lost health are immense. The risk of a worse health outcome such as chronicity increases if general depression is not timely treated (Ghio, Gotelli, Marcenaro, Amore, & Natta, 2014) and lower economic production is present (Chisholm et al., 2016). Although effective methods for the prevention and treatment of depression symptoms exist, the vast majority of people with general depressive symptoms do not receive any treatment (Patel, 2017).

Symptoms of anxiety and depression are more prevalent among women than men (WHO 2017), with a number of conditions that can lead to these states such as poverty (Lund et al., 2010) and unemployment (Heinz et al., 2018), and others that seem protective, like cohabitation (Van de Velde, Bracke, & Levecque, 2010). The symptoms are also comorbid in a number of chronic diseases such as respiratory disorders, coronary heart disease and diabetes (Guthrie et al., 2016), with the prevalence of psychological disturbances among people increasing with the number of chronic disorders they have (Barnett et al., 2012).

#### 1.1.1 Anxiety and depression in surgical populations

A literature search revealed that the majority of studies on anxiety and depression symptoms related to surgery have been conducted among heart surgery patients followed by orthopaedic surgery patients. The studies report and compare pre‐ and postoperative conditions. However, timing may differ between studies from months to a day before the operation and from 6 days until a year after the operation. Definitions of anxiety and depression may also differ between studies as a result of different instruments being used to measure symptoms. In some studies, the authors are screening for symptoms of anxiety or depression, while in others, they are using a diagnosis of anxiety or depression. Given these precautions, the prevalence of pre‐operative symptoms of anxiety has been reported as 15%–52% (Tully, Cosh, & Baumeister, 2014) and 10%–60% of depression symptoms (Blumenthal et al., 2003; Duivenvoorden et al., 2013). Studies on the course of these disturbances are not univocal. Anxiety has been reported to be generally low and stable over time (Gallagher & McKinley, 2009; Sveinsdóttir & Ingadóttir, 2012; Sveinsdóttir & Skúladóttir, 2012), but also highest before surgery, decreasing immediately after surgery but constant up to 6 months postoperatively (Duits et al., 1998; McCrone, Lenz, Tarzian, & Perkins, 2001; Taillefer et al., 2006). Studies on the course of depression symptoms show an increase from before discharge from hospital to 2 weeks postdischarge (Gallagher & McKinley, 2009); a decrease from surgery to 4 weeks postsurgery (Sveinsdóttir & Ingadóttir, 2012; Taillefer et al., 2006); an occasional prior to surgery, peaking at day two or three postsurgery, decreasing again 2 weeks postsurgery and remaining constant until 12 weeks postsurgery (McCrone et al., 2001); and an increase postsurgery (Nickinson, Board, & Kay, 2009; Sveinsdóttir & Skúladóttir, 2012). Rothenhausler et al. (2005) studied the natural history of psychiatric morbidity for 1 year postheart surgery and found short‐term recovery to be associated with symptoms of depression, although at 12 months, anxiety and depression had returned to pre‐operative level.

Studies on the influence of pre‐operative anxiety and depression symptoms on outcomes have found these disturbances to be associated with more severe physical symptoms, hospital readmissions, longer hospital stay, increased cardiac events, worse general function, pain, lower quality of life and various postoperative complications (Blumenthal et al., 2003; Faller, Kirschner, & Konig, 2003; Geulayov et al., 2018; Gold et al., 2016; Hanusch, O'Connor, Ions, Scott, & Gregg, 2014; Kagan & Bar‐Tal, 2008; Poole et al., 2014, 2017; Rasouli, Menendez, Sayadipour, Purtill, & Parvizi, 2016; Rolfson, Dahlberg, Nilsson, Malchau, & Garellick, 2009; Stark et al., 2016; Tully et al., 2014, 2015). The presence of symptoms of anxiety or depression at the time of surgery, along with pain and not feeling rested upon awakening, seems to be a major contributor to the presence of those same symptoms postsurgery (Basak et al., 2015; Sveinsdóttir, 2010).

A recent systematic review and meta‐analysis of the association between pre‐operative depression and long‐term survival following coronary artery bypass surgery reported an increase in all‐cause mortality in relation to pre‐operative depression in four out of seven studies that were included in the analysis (Stenman et al., 2016). Also, in support of the influence of psychosocial well‐being on outcomes, a 5‐year longitudinal study found generalized anxiety disorder to be significantly associated with major adverse cardiovascular and cerebrovascular events (Tully et al., 2015). A systematic review and meta‐analysis of the effect of pre‐operative education on postoperative anxiety among cardiac surgery patients found the intervention to effectively decrease anxiety in this patient group (Ramesh et al., 2017).

As mentioned, the main focus of perioperative care is patient safety through pre‐operative evaluation and interventions to ensure optimal condition before surgery and to support postoperative recovery. Here, nurses play an important role as by delivering patient education and providing direct physical care. The summary above shows that the focus should also be directed to the presence of symptoms of anxiety and depression. In the present study, we use the Hospital Anxiety and Depression Scale (HADS) as an outcome measure of surgical patients' psychological well‐being and various situations that are related to symptoms of anxiety or depression as independent measures that may influence the presence of anxiety or depression up to 6 months postdischarge from hospital. The specific aims of the study are to (a) describe prevalence of symptoms of anxiety and depression in surgical patients at three time points: at hospital postsurgery, 6 weeks and 6 months postdischarge from hospital; (b) detect situations and experiences that predict symptoms of anxiety and depression six weeks and 6 months postdischarge.

## 2 METHODS

### 2.1 Research design and setting

This was a prospective, explorative two‐site follow‐up study. The setting was the two university hospitals in the country (a) the Landspitali University Hospital (LUH), a tertiary university hospital that has 669 beds and a mean length of stay at the surgical division of 5.0 days and that performs all major surgeries (Landspítali, 2017) and (b) the Akureyri Hospital (AH), a teaching hospital that has 110 beds and mean length of stay at the surgical division of 2.9 days and that performs mostly hip and knee replacements (Sjúkrahúsið á Akureyri, 2017).

### 2.2 Participants and procedure

Eligible for participation were patients who had elective cardiac or pulmonary surgery or urological, gastrointestinal or orthopaedic surgery (knee or hip replacement) at LUH and orthopaedic surgery (knee or hip replacement) at AH from 15 January–15 July 2016, who could read and write Icelandic, who stayed at the hospital overnight, who were discharged home, who were at home six weeks and 6 months postdischarge, and who were assessed by nurses as eligible for participation based on the patients' mental capacity and degree of physical state at the time. This assessment was based on a discussion and evaluation of the patient. If the nurse discovered, while talking to the patient, that he had problems understanding the purpose of the study or the questions, he stated he did not feel up to participation, or if the patient was obviously very sick, he was non‐eligible for participation. Data collection was completed 6 months after the last patient was discharged from hospital. Participation included answering questionnaires at the hospital postsurgery (T1), 6 weeks postdischarge (T2) and 6 months (T3) postdischarge.

During hospital admittance, the patients were approached by the nurse unit manager or her/his representative who introduced the study and asked if a study nurse might contact them on the ward. If the patient agreed, the nurse explained the study on postoperative day 1, 2 or 3 depending on type of operation and the patient's condition and gave him/her the hospital questionnaire to fill out. All participants answered the hospital questionnaire in a paper and pencil format but could choose paper or online format for the other questionnaires. Online data were collected and managed using REDCap (nd) electronic data capture tools hosted at the University of Iceland's School of Health Sciences. Participants received the paper version of the home questionnaires by post or the online version by email, with a reminder sent via text messaging and email twice after the questionnaire was sent. The procedure was repeated once for those who had not answered two weeks after the initial due date.

**FIGURE 1:**
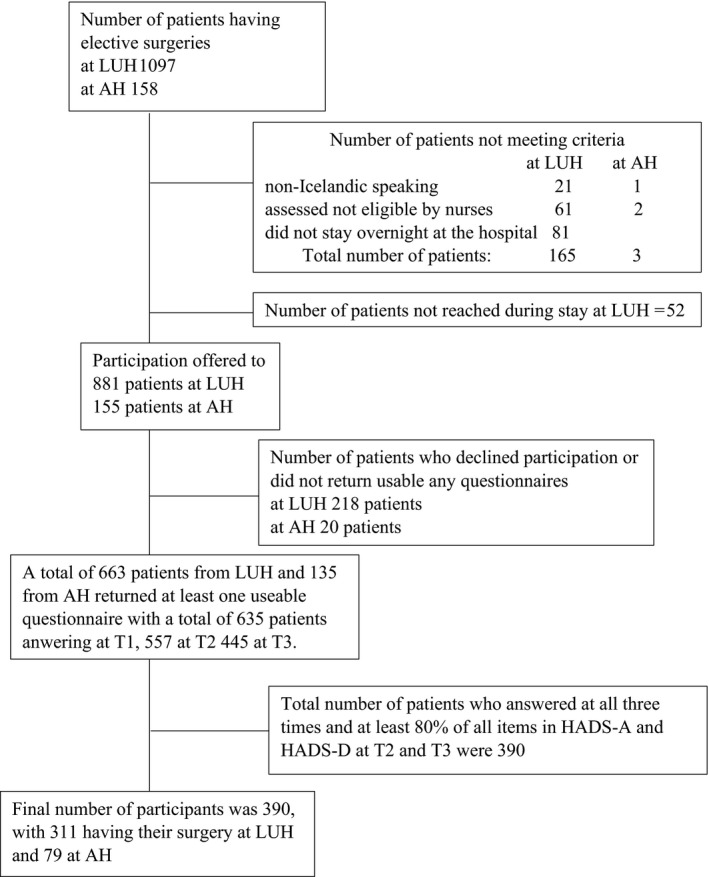
Flowsheet of patient participation in the study by Landspítali University (LUH) and Akureyri Hospital (AH)

During the study period, 1,097 patients at LUH and 158 at AH underwent the elective surgeries previously defined. Figure 1 presents a flowsheet of study participation. Just over half of the patients chose to answer online at T2 and T3. Their response rate was higher at both T2 and T3 than the response rate of those answering in paper and pencil format (79% versus 59% at T2 and 74% versus 45% at T3).

Since one of the main aims was to predict anxiety and depression as measured by HADS six weeks and 6 months postdischarge, only patients who answered at least 80% of all items in HADS‐A and HADS‐D at those times were included, resulting in a total of 390 participants (see Figure 1).

### 2.3 Data collection

#### 2.3.1 Symptoms of anxiety and depression—outcome variables

Symptoms of anxiety and depression were measured, at all times, with the HADS that was developed with the purpose of screening for symptoms of anxiety and depression among non‐psychiatric hospitalized patients (Zigmond and Snaith, 1983). HADS consists of two independent subscales, HADS‐A (symptoms of anxiety) and HADS‐D (symptoms of depression), each with seven items that measure symptoms of anxiety and depression, respectively. Each item is rated on a scale from 0–3 with the possible total scores from each subscale ranging from 0–21. Those who score 8–10 are categorized as having moderate symptoms of depression and/or anxiety and those who score 11–21 as having severe symptoms. HADS has been standardized for use in Iceland (Smári et al. 2008), and in this study, Cronbach's alpha for HADS‐A at T1, T2 and T3 was 0.87, 0.86 and 0.87 respectively. For HADS‐D, it was 0.77, 0.82 and 0.83, respectively.

#### 2.3.2 Predictor variables

The predictor variables are experiences and situations that are present during the postoperative recovery and have been found to be associated with symptoms of anxiety or depression. In this study, these are variables addressing symptoms, sleep, comorbidities, recovery, work, length of stay, patient education and background variables.

Patients were asked about distress caused by 14 common postoperative symptoms (excluding pain): diarrhoea, nausea, constipation, vomiting, difficulty with initiating voiding, urinary incontinence, impaired memory, trouble with sexual activities, trouble with movement, loss of appetite, weakness, tiredness, sleeplessness and dyspnoea; measured on a scale from 1 (no distress at all)–5 (very much distress) 7 days prior to hospitalization (T1) and during the 7 days before the questionnaire was answered at T2 and T3. In the present study, we decided to average the scores of all the symptoms at T2 and all the symptoms at T3 into "Postoperative Symptoms DistressT2" and "Postoperative Symptoms DistressT3" and use as overall indicators for postoperative symptoms of distress at those two time points. Higher scores indicated more distress. Reliability testing found Cronbach's alpha in this study to be 0.81 for SymptomDistressT2 and 0.83 for SymptomDistressT3.

Pain was assessed at all times with questions about the presence of pain during the past 24 hr (yes/no) and what the average pain rating was on a scale from 0 (no pain)–10 (worst imaginable pain). At T2 and T3, patients were asked if they had experienced pain related to their surgery since they were discharged from hospital, and, if so, whether the pain was experienced constantly, often or sometimes. Findings on pain and postoperative symptom distress are reported for T2 and T3 only.

Sleep was assessed at T2 and T3 by asking how often (never/less than once a week/1–2 times a week/daily) the patient did not feel fully rested upon awakening. The presence of comorbidities was asked about at T1 (responses yes/no), if patients answered yes, they were asked if they had any of the following diseases: hypertension, arthritis, cardiovascular disease, cancer, diabetes, pulmonary disease or mental illness. Participants could mark more than one disease. We did not have access to any of the patients' diagnosis in their medical charts including psychiatric diagnosis. Recovery and patient education were assessed at T2 and T3 by asking patients how well they have recovered since the operation (very well/well/fairly well/badly/very badly) and how useful they found the discharge education they received (very useful/rather/neither or/little/not useful).

Background information such as age, gender, living with spouse at home (yes/no), children living at home (yes/no) was asked for at T1, while at T2 and T3, patients were asked if they had resumed work.

Information about type of operation and length of stay (LOS) was retrieved from patient records.

### 2.4 Ethics

Participants received a letter explaining the purpose of the study, what participation involved and the questionnaire. They were informed about their rights to withdraw from the study whenever they chose to do so. The letter also included information on responsible parties and on contact persons should they have any questions or comments. Patients who agreed to participate in the study signed an informed consent and received further explanation of the study on the hospital ward after surgery. The study was approved by the National Bioethics Committee (registration number 15‐040‐V1) and the directors of the surgical division at both hospitals as required by law.

### 2.5 Data analysis

Statistical analyses were carried out using Statistical Package for Social Sciences, 24.00 (IBM Corp. 2016). Missing data were handled by pairwise deletion.

All categorical variables were decoded as dichotomous and presented as such. Descriptive data are presented as mean values, with standard deviations and percentages. Relationships between outcome variables and predictor variables are presented using *t* test for continuous variables and chi‐square for categorical ones, and ANOVA was used to test for differences in mean scores by surgeries. The significance level was set at < 0.05. A stepwise multiple linear regression model was employed to calculate predictors of the mean score of HADS‐D and HADS‐A at T2 and T3. For each model, variables (shown in Table 1) were entered in three steps arguably reflecting a causal order. The enter method was used within steps. Variables entered in the models differed for models at different times (see specific variables for each model in Tables 3 and 4). In general, in step one background variables were entered, in step two variables related to surgery, diseases, hospitalization, patient education, recovery and sleep, and in step three symptom variables (pain, postoperative symptom distress and HADS).

**Table 1:** Descriptive characteristics of participant (*N* = 390) at hospital, six weeks (T2) and 6 months (T3) postdischarge and significant relationships with HADS‐A^a^ and HADS‐D scores at those times

Continuous variables^c^	*n*	Mean (*SD*)	HADS‐A T2	HADS‐A T3	HADS‐D T2	HADS‐D T3
			p.	p.	p.	p.
Age (mean ± *SD*)	342	63.9 (11.7)	<.01	<.01	ns	ns
HADS‐A at T1^b^	347	5.1 (4.0)	<.01	<.01	<.01	<.01
HASD‐A at T2	372	3.8 (3.5)	–	<.01	<.01	<.01
HADS‐A at T3	376	4.1 (3.6)	<.01	–	<.01	<.01
HADS‐D at T1	350	2.6 (2.7)	<.01	<.01	<.01	<.01
HADS‐D at T2	378	2.8 (3.0)	<.01	<.01	–	<.01
HADS‐D at T3	378	3.1 (3.2)	<.01	<.01	<.01	–
Mean intensity of pain during the last 24 hr at T2^b^	200	3.1 (2.0)	<.01	<.01	–	<.01
Mean intensity of pain during the last 24 hr at T3	147	3.1 (1.9)	<.01	<.01	–	<.01
Postoperative symptoms distress T2^b^	280	1.5 (0.4)	<.01	<.01	<.01	<.01
Postoperative symptoms distress T3	283	1.4 (0.4)	<.01	<.01	<.01	<.01
Length of stay (days)	390	4.0 (3.1)	ns	<.05	ns	<.01

Categorical variables^d^	*n* (%)				
Gender (male) (N^e^ = 344)	173 (50.3)	ns	ns	ns	ns
Living with spouse at home (yes) (*N* = 346)	276 (79.8)	ns	ns	ns	ns
Children living at home (yes) (*N* = 347)	44 (12.7)	<.05	ns	ns	ns
Experiencing pain last 24 hr at T2 (yes) (*N* = 304)	168 (55.3)	<.01	<.05	<.05	<.01
Experiencing pain last 24 hr at T3 (yes) (*N* = 246)	116 (47.2)	<.01	<.01	<.01	<.01
Recovery good/very good at T2 (*N* = 389)	270 (69.4)	<.01	<.01	<.01	<.01
Recovery good/very good at T3 (*N* = 380)	271 (80.3)	<.01	<.01	<.01	<.01
Other diseases (yes) (*N* = 339)	269 (79.4)	ns	ns	<.01	<.05
Patient education very useful at T2 (*N* = 375)	230 (61.3)	<.01	<.01	<.01	<.01
Patient education very useful at T3 (*N* = 380)	167 (43.9)	<.05	<.01	<.05	<.01
Has begun working at T2 (*N* = 385)	79 (20.5)	ns	ns	ns	ns
Has begun working at T3 (*N* = 385)	178 (46.2)	ns	ns	<.01	<.01
Fully rested upon awakening once, twice a week or daily at T2 (*N* = 348)	170 (48.9)	<.01	<.01	<.01	<.01
Fully rested upon awakening once, twice a week or daily at T3 (*N* = 350)	156 (44.5)	<.01	<.01	<.01	<.01

^a^ HADS‐A Hospital and Anxiety Scale‐Anxiety, HADS‐D Hospital and Anxiety Scale‐Depression.

^b^ Scores on HADS subscales range from 0–21 with higher scores indicating more symptoms of anxiety or depression; pain scores range from 0–10 with higher scores indicating more pain; and scores on Postoperative Symptom Distress range from 1–5.

^c^ Significance based on Pearson's correlations.

^d^ Significance based on *t* test.

^e^ *N* indicates number of participants answering the questions. Valid percentage is presented.

Assumptions of regression related to multicollinearity by the use of the variance inflation factor (VIF) method and correlation between independent variables were examined (Field, 2013).

A prior power analysis was conducted to estimate sample size for multiple regression using GPower (Erdfelder, Faul, & Buchner, 1996). The criterion for statistical significance was set at *α* = 0.05, two‐tailed, power (1−*β*) was set at 0.80, effect size was set at 0.08, and 16 predictors were assumed. This showed us that in order to reach statistical significance at the 0.05 level our sample size was estimated to be 255, respectively.

## 3 RESULTS

### 3.1 Descriptive findings

The majority of patients had orthopaedic surgery (*n* = 233), followed by gastrointestinal (*n* = 78), urological (*n* = 41) and thoracic (cardiac or pulmonary) (*n* = 38) surgery.

#### 3.1.1 Symptoms of anxiety and depression

Mean score on HADS‐A for all patients having all surgeries was highest at hospital (*M* = 5.1, *SD* = 4.0), lowest at six weeks (*M* = 3.8; *SD* = 3.5) and higher again at 6 months postdischarge (*M* = 4.1; *SD* = 3.6). For HADS‐D, the scores increased from hospital (*M* = 2.6; *SD* = 2.7), to six weeks (*M* = 2.8, *SD* = 3.0) and to 6 months postdischarge (*M* = 3.1; *SD* = 3.2) (Table 1). For individual surgeries, the highest mean HADS‐A score was among patients having general surgery at the hospital (*M* = 6.4; *SD* = 4.7) and lowest among patients having orthopaedic surgery six weeks postdischarge (*M* = 3.5; *SD* = 3.2). The highest mean HADS‐D score was found among patients having thoracic surgery six weeks postdischarge (*M* = 3.9; *SD* = 3.5) and the lowest mean score among patients having urological surgery at the hospital (*M* = 2.4; *SD* = 2.9). A one‐way ANOVA (not shown in tables) was conducted in order to detect significant differences by surgeries in mean scores on HADS‐A and HADS‐D at different times. A Tukey post hoc test revealed significant difference once: at T1 patients having general surgery scored higher on HADS‐A (*M* = 6.4, *SD* = 4.7) as compared with patients having orthopaedic surgery (*M* = 4.7; *SD* = 3.5).

**FIGURE 2:**
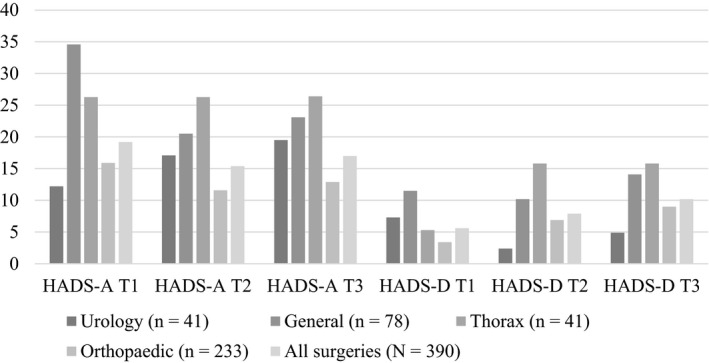
Percentage of patients presenting with moderate or significant anxiety or depression at the hospital (T1), 6 weeks (T2) and 6 months postdischarge (T3) by surgeries

Table 2 shows number of patients presenting with normal, moderate and severe symptoms of anxiety and depression. It can be seen that at hospital 20.2% (*n* = 79) and at 6 months postdischarge 17.0% of patients (*n* = 66) experienced moderate‐to‐severe symptoms of anxiety and for depression the numbers are 5.6% (*n* = 22) at hospital and 10.2% (*n* = 40) at 6 months postdischarge. For comparison by surgeries, Figure 2 displays the proportion of patients presenting with moderate‐to‐significant anxiety or depression by surgeries. Significant difference is not reported. The actual number of patients is low for each surgery, but the figure shows the trend in progression of the symptoms over time. The figure shows that the highest proportion of patients having general surgery experience such anxiety at the hospital, then the numbers drop 6 weeks postdischarge and are similar 6 months postdischarge. The proportion of patients having thoracic surgery stays the same at all time points and of those having urology surgery increasing a little. The proportion of all patients' groups, except urology patients, experiencing such depression increases from T1–T3.

**Table 2:** Number of patients presenting with, normal, moderate and severe symptoms of anxiety and depression at all times

	At the hospital (T1)		At home six weeks postdischarge (T2)		At home 6 months postdischarge (T3)	
	Anxiety	Depression	Anxiety	Depression	Anxiety	Depression
	*n* (%)	*n* (%)	*n* (%)	*n* (%)	*n* (%)	*n* (%)
Normal	267 (68.5)	328 (84.1)	312 (80)	357 (89.0)	310 (79.5)	338 (86.7)
Moderate	36 (9.2)	16 (4.1)	42 (10.8)	22 (5.6)	42 (10.8)	20 (5.1)
Severe	43 (11.0)	6 (1.5)	18 (4.6)	9 (2.3)	24 (6.2)	20 (5.1)

^a^ Possible total scores range from 0–21. Those who score above 10 are categorized as having moderate‐to‐severe symptoms of depression and/or anxiety.

#### 3.1.2 Predictor variables

Descriptive findings are presented in Table 1. The participants ranged in age from 18–88 years (mean = 63.9 years; *SD* = 11.7 years). The gender ratio was almost equal. A majority lived with a spouse (79.8%) and with no children (87.3) at home. At T2, 20.5% had resumed work and at T3, 46.2%. Average LOS was 4.0 days (*SD* = 3.1; range = 0.6–20.9 days), and 79.4% had some comorbidities, including hypertension (40%), arthritis (29.5%), cardiovascular disease (17.7%) and mental illness (4.4%). A majority of patients had experienced surgery‐related pain at T2 (74.2%) and T3 (58.7%), with 14.4% having experienced constant pain since the operation at T2 and 8.8% at T3 (not shown in table). A minority felt fully rested upon awakening at T2 (48.9%) and T3 (44.5%). The mean score on SymptomDistressT2 was 1.5 (*SD* = 0.4 range 1–3.2) and on SymptomDistressT3 1.4 (*SD* = 0.4 range 1–3.2).

### 3.2 Relationships between outcome variables and predictor variables at T2 and T3

It can be seen from Table 1 that in general for significant associations, higher scores on HADS‐A and HADS‐D at one time point correlate with higher scores on the scales at other time points. The same applies for severity of pain, postoperative symptom distress and LOS—that is, the greater the severity or distress or the longer the LOS the higher the scores were on HADS‐A and HADS‐D. Patients who at T2 and T3 experienced no pain during the last 24 hr, who reported good recovery, found education very useful and were fully rested upon awakening once or twice a week scored lower on HADS‐A and HADS‐D at both times. Finally, patients with no comorbidities and those who had resumed work scored lower on HADS‐D at T2 and T3.

### 3.3 Predictors of symptoms of anxiety and depression as measured by HADS‐D and HADS‐A

Based on the findings that not many differences in mean HADS‐A and HADS‐D scores at different times by type of surgery existed, we decided not to use type of surgery in the regression analysis but to implement LOS as a proxy for severity of illness.

Correlation between the outcome variables and independent variables was mostly low. VIF was in most instances between 1.0–1.4 but was over 2 for HADS‐D T3 with HADS‐D T2 (VIF = 2.022) and HADS‐A T2 (VIF = 2.132). This indicates that the data met criteria for the non‐existence of multicollinearity.

#### 3.3.1 Predictors of symptoms of anxiety as measured by HADS‐A six weeks (T2) and 6 months (T3) postdischarge from hospital

There are three successive models presented for T2 and T3. At T2 (Table 3), the explained variance increased from 6.0% in model 1%–50.9% in model 3 and at T3 (Table 4) from 7.1% in model 1%–55.5% in model 3. While the contribution of each variable was not always statistically significant, the results suggest that the variables in the model contribute to scores on HADS‐A at T2 and T3. However, model 3 in the T2 models showed that, holding other independent variables constant, the strongest predictor for higher scores on HADS‐A (indicating more symptoms of anxiety) was experiencing more distressing postoperative symptoms at T2 (*b* = 1.76), followed by not feeling rested upon awakening at T2 (*b* = 0.90) and higher scores on HADS‐A at T1 (*b* = 0.38). At T3, the strongest predictors as shown by model 3 were not feeling rested upon awakening at T3 (*b* = 1.20) and higher scores on HADS‐A at T2 (*b* = 0.59).

**Table 3:** Regression models for potential predictors of HADS‐A and HADS‐D scores six weeks postdischarge from hospital

Variables	Anxiety 6 weeks postdischarge						Depression 6 weeks postdischarge					
	Model 1		Model 2		Model 3		Model 1		Model 2		Model 3	
	B	*t*	B	*t*	B	*t*	B	*t*	B	*t*	B	*t*
(Constant)	7.41	4.31***	5.02	2.96***	−0.16	−0.08	3.39	2.25*	1.51	0.99	−2.61	−1.48
Step 1 background variables												
Age (mean + *SD*)	−0.06	−2.50**	−0.06	−2.49**	−0.02	−0.94	−0.01	−0.38	−0.01	−0.24	0.01	0.40
Gender (male)	0.60	1.17	0.39	0.81	−0.34	−0.84	0.19	0.42	−0.01	−0.02	−0.32	−0.86
Living with spouse at home (yes)	0.42	0.59	0.56	0.85	0.48	0.91	0.18	0.29	0.26	0.44	0.20	0.42
Children living at home (yes)	0.24	0.26	0.31	0.35	0.45	0.64	−0.09	−0.11	0.09	0.12	0.08	0.12
Has begun working at T2	0.14	0.25	0.86	1.61	0.18	0.41	−0.34	−0.72	0.23	0.49	−0.39	−0.95
Step 2 variables measuring other diseases, length of stay, recovery, sleep and patient education												
Other diseases (yes)			0.65	1.05	0.08	0.16			0.80	1.44	0.39	0.86
Length of stay (days)			0.08	1.07	0.03	0.48			0.09	1.29	0.02	0.27
Recovery good/very good at T2			−1.73	−3.20***	−0.64	−1.36			−1.33	−2.75**	−0.37	−0.87
Fully rested upon awakening almost daily at T2			−1.88	−3.88***	−0.90	−2.22*			−1.20	−2.76**	−0.58	−1.55
Patient education very useful at T2			−0.61	−1.24	−0.32	−0.79			−0.75	−1.69	−0.48	−1.32
Step 3 variables measuring pain, physical symptoms and symptoms of anxiety and depression												
Experiencing pain last 24 hr at T2					−0.25	−0.56					0.21	0.53
Postoperative symptom distress T2					1.76	2.83**					2.15	3.81***
HADS‐D at T1					0.15	1.60					0.44	5.12***
HADS‐A at T1					0.38	5.74***					0.03	0.47
*R* ^2^	.060*		.227***		.509***		.006		.146***		.439***	
*F*	2.273		5.091		12.519		0.192		2.961		9.459	

Abbreviations: HADS‐A Hospital and Anxiety Scale‐Anxiety; HADS‐D, Hospital and Anxiety Scale‐Depression.

* *p* < .05.

** *p* < .01.

*** *p* < .001.

#### 3.3.2 Predictors of symptoms of depression as measured by HADS‐D 6 weeks (T2) and 6 months (T3) postdischarge from hospital

As regards depression, there were three successive models presented for T2 (Table 3) and T3 (Table 4). At T2, the explained variance increased from 0.6% in model 1%–43.9% in model 3 and at T3 from 5.1% in model 1%–55.6% in model 3. Also, while the contribution of each variable was not always statistically significant, the results suggest that the variables in the model contribute to scores on HADS‐D at T2 and T3. Model 3 in the T2 model showed that, holding other independent variables constant, the strongest predictor for higher scores on HADS‐D (indicating more symptoms of depression) was experiencing postoperative symptoms as more burdensome at T2 (*b* = 2.15), followed by higher scores on HADS‐D at T1 (*b* = 0.44). At T3, the strongest predictor (as shown by model 3) is also experiencing more distressing postoperative symptoms at T2 (*b* = 1.29), followed by delayed or very delayed recovery from the operation at T3 (*b* = −1.26) and higher scores on HADS‐D at T2 (*b* = 0.53).

**Table 4:** Regression models for potential predictors of HADS‐A and HADS‐D scores 6 months postdischarge from hospital

Variables	Anxiety 6 months postdischarge						Depression six months post discharge					
	Model 1		Model 2		Model 3		Model 1		Model 2		Model 3	
	B	*t*	B	*t*	B	*t*	B	*t*	B	*t*	B	*t*
(Constant)	9.69	4.57***	5.11	2.43*	0.66	0.32	6.31	3.31***	2.09	1.12	−0.81	−0.44
Step 1 background variables												
Age (mean + sd)	−0.08	−2.62*	−0.06	−2.03*	−0.02	−0.99	−0.03	−1.07	−0.01	−0.45	0.00	−0.15
Gender (male)	0.42	0.70	0.14	0.26	−0.07	−0.15	−0.10	−0.18	−0.32	−0.67	−0.30	−0.78
Living with spouse at home (yes)	0.49	0.61	0.55	0.74	0.27	0.47	0.26	0.36	0.32	0.48	0.18	0.34
Children living at home (yes)	−0.31	−0.29	0.21	0.21	−0.16	−0.21	−0.50	−0.52	0.01	0.01	−0.14	−0.20
Has begun working at T3	−1.19	−1.61	−0.20	−0.28	−0.10	−0.18	−1.74	−2.62*	−0.57	−0.90	−0.08	−0.15
Step 2 variables measuring other diseases, length of stay, recovery, sleep and patient education												
Other diseases (yes)			1.19	1.71	0.42	0.76			0.90	1.46	0.12	0.24
Length of stay (days)			0.07	0.78	0.03	0.49			0.08	1.02	0.03	0.46
Recovery good/very good at T3			−1.58	−2.40*	−0.65	−1.12			−2.24	−3.84***	−1.26	−2.42*
Fully rested upon awakening almost daily at T3			−2.35	−4.16***	−1.20	−2.57*			−1.68	−3.35***	−0.68	−1.63
Patient education very useful at T3			−0.42	−0.75	−0.15	−0.33			−0.43	−0.87	−0.14	−0.34
Step 3 variables measuring pain, physical symptoms and symptoms of anxiety and depression												
Experiencing pain last 24 hr at T3					0.19	0.38					−0.02	−0.04
Postoperative symptom distress T3					0.93	1.37					1.29	2.14*
HADS‐D at T2					−0.01	−0.15					0.53	6.03***
HASD‐A at T2					0.59	6.83***					0.04	0.51
*R* ^2^	0.071		0.261***		0.555***		0.051		0.269***		0.556***	
*F*	2.175		4.486		11.939		1.521		5.077		11.977	

Abbreviations: HADS‐A, Hospital and Anxiety Scale‐Anxiety; HADS‐D, Hospital and Anxiety Scale‐Depression.

* *p* < .05.

*** *p* < .001.

## 4 DISCUSSION

The main focus of nursing care of surgical patients has been on physical care. The findings of this study are that a considerable proportion of surgical patients' experience symptoms of anxiety and depression, as measured with HADS, both at hospital and up to 6 months postdischarge. In addition, our findings confirm earlier findings that having symptoms of anxiety or depression predicts symptoms of anxiety and depression (Sveinsdóttir, 2010; Sveinsdóttir & Ingadóttir, 2012; Sveinsdóttir & Skúladóttir, 2012), indicating that psychological care is of utmost importance.

Interestingly, there was not much difference in mean scores of anxiety and depression scores by type of surgery, which supports the view that surgery in and of itself influences psychological well‐being. Our findings indicate that the total number of patients experiencing moderate to significant anxiety decreases from postsurgery at the hospital until 6 months later, while numbers for patients experiencing moderate‐to‐severe depression between those time points almost doubled. The same applied for mean scores on HADS‐A from T1–T3,—that is they decreased—and the mean scores on HADS‐D increased. We did not test for statistical differences by surgery types between the number of patients experiencing moderate or significant anxiety or depression at different times. Our analysis of the number of patients presenting with such anxiety or depression by surgery type suggests that the trend over time may differ by surgery type. However, for a valid comparison a larger sample is needed.

The average score on the HADS‐scales in our study was comparable to previous Icelandic studies on patient populations, that is surgical patients (Sveinsdóttir & Ingadóttir, 2012; Sveinsdóttir & Skúladóttir, 2012) and heart patients (Ketilsdottir et al., 2019); and among university students (Smári et al. 2008). Compared to global prevalence of depressive disorder and anxiety Iceland lies close to the average, with 4.1% prevalence of depression and 4.9% of anxiety compared to 4.4% and 3.6% globally respectively (WHO, 2017).

Surprisingly, as described in the introduction, we did not find many studies that have addressed the course of anxiety and depression over time postsurgery. The results of those we found were not equivocal, and no study was found where depression increased over a 6‐month period as in our study. In order to determine the course of postsurgery depression, this finding warrants further studies in larger patient populations. Studies on the course of anxiety showed results similar to those of our study (McCrone et al., 2001; Taillefer et al., 2006).

Distress caused by postoperative symptoms was the strongest predictor of symptoms of anxiety six weeks postsurgery and symptoms of depression six weeks and 6 months postsurgery. In addition, similar to earlier findings (Sveinsdóttir, 2010; Sveinsdóttir & Ingadóttir, 2012; Sveinsdóttir & Skúladóttir, 2012), more symptoms of anxiety or depression at one time point predict more symptoms at the next time point. A study on depression screening after cardiac surgery found that patients meeting screening criteria for depression at the hospital presented with a higher risk of major adverse cardiac event, depressive mood and having started antidepressants 12 months postdischarge (Tully, Baumeister, Bennets, Rice, & Baker, 2016). Another study that focused on anxiety and depression at 12 months postcardiac surgery found anxiety symptoms to be associated with greater pain and postoperative symptoms 12 months postsurgery (Poole et al., 2017). Taken together, these findings suggest that it is good clinical practice to screen patients for symptoms of depression and anxiety pre‐operatively and before discharge and to follow‐up those who meet the screening criteria—for the HADS‐scales scoring above 10 ‐during the recovery period. In the present study, the total number of patients is approximately one hundred, a large number in a small population like Iceland. Keeping in mind the adverse health (Ghio et al. 2014) and economic (Chisholm et al., 2016) outcome if general depression is not timely treated the benefits from a follow‐up may be substantial in terms of decreased suffering, medical cost and even chronic disability. As postoperative symptom distress predicted both anxiety and depression, patients may benefit from symptom management aimed at reducing symptom distress. The same holds true for improving sleep and reducing anxiety in the postoperative period and after discharge. Research indicates that the relationship between sleep deprivation and anxiety may be bi‐directional—that is, anxiety disorder generates sleep loss and perhaps insomnia, and sleep deprivation leads to anxiety (Pires, Bezerra, Tufik, & Andersen, 2016). The hospital environment in the immediate postoperative period then contributes to sleep disturbances where sleeping in room with other patients and common night‐time care routines, such as assessment of the patients, administration of medication and laboratory tests, performed by nurses influence night‐time sleep effectiveness (Casida, Davis, Zalewski, & Yang, 2018). Although sleep was not assessed at the hospital in our study, our findings indicate that patients should be screened for sleep deprivation during follow‐up appointments.

Experiencing unsuccessful recovery 6 months postsurgery predicted symptoms of depression at that time. This may be understandable, for example when patients undergo surgery for incurable gastrointestinal cancer and when full recovery cannot be expected. That was not the situation of the majority of participants. Studies have shown that psychological health influences symptoms postoperatively. A systematic review found that pre‐operative pain catastrophizing was related to more severe postoperative pain and that worse mental health, pre‐operatively, resulted in more pain and diminished function up to 1 year following orthopaedic surgery (Vissers et al., 2012). This indicates that patients with unsuccessful recovery should be followed up about psychological distress.

Comorbidities did not significantly influence symptoms of anxiety or depression, as may be expected. Although we did not correct for the number of comorbidities, this may be an important issue to look at—for example, if the patient has both arthritis and diabetes. Information on comorbidities was gained from the patients themselves. It might have been more precise to retrieve this information from the patients' medical record. However, gaining them from patients is hardly problematic since nurses assessed the mental capacity of all patients during recruitment.

Our finding that patient education correlated with less anxiety and depression supports Ramesh et al. (2017) on the effectiveness of pre‐operative education on postoperative anxiety. Likewise, our findings that less pain, having no other diseases and having resumed work are associated with less anxiety or depression are in line with earlier findings (Poole et al., 2017; Sveinsdóttir & Ingadóttir, 2012; Sveinsdóttir & Skúladóttir, 2012). Nurses working on surgical units should be aware of this, and pain management and good sleep at night should be prominent in their pre‐operative education. Furthermore, it should be anticipated that patients with comorbidities receive more thorough follow‐up.

Interestingly, gender was not a predictor in any of our models. A systematic review on CABG patients found studies to be inconclusive in this matter (McKenzie, Simpson, & Stewart, 2010). They suggest that reasons for inconclusiveness postoperatively in general might relate to gender susceptibility to poor health, socio‐economic status and social support. In this study, our focus was not on gender; however, it would be interesting to study further the resolution of psychological symptoms postoperatively based on gender.

Finally, to self‐report symptoms of anxiety or depression is not the same as being diagnosed with depression or anxiety disorder. The symptoms themselves are a part of people's daily living and assist in facing daily challenges and difficult situations such as surgery. However, when prolonged, they can develop into disorders that have adverse health effects. Therefore, though not diagnostic in themselves, patients' self‐reported symptoms are of when diagnosing anxiety/depressive disorders, and when monitoring treatment.

### 4.1 Limitations

In the regression, we did not differentiate between severity of surgery but used length of hospital stay as a proxy for this differentiation. Length of stay and type of surgery both represent the severity of the underlying illness and yet obviously severe complications leading to a longer stay can follow any surgery. We adjusted for comorbidities but acknowledge that different diseases, such as cancer, can have different significance. We were interested in analysing total postoperative symptom distress but are aware that it may be questionable to group the 14 symptoms together and average the scores. In addition, the questions on pain may not have adequately captured the severity of pain as patients were asked to report on their average pain. A question on time spent in severe pain would have been of benefit. We decided to use HADS at all time points despite the fact that it was developed for use among hospitalized patients. As the Icelandic version of HADS has demonstrated good psychometric properties (Smári et al. 2008) and the questions reflect a good index for psychological distress, we deemed them as adequate for use for posthospitalization also. It should also be acknowledged that one of the inclusion criteria is based on nurses' clinical assessment; therefore, it might be subjective. When designing the study, however, we concluded that the nurses were qualified to make this assessment based on their clinical knowledge. A major strength of this study is that all patients having elective surgery at the time of the study were invited to participate. However, the small size of patients answering at all three times is a limitation and we do not have data to assess how well our sample represents the population. The patients were asked to answer the questionnaire on days one to three postsurgery. We did not ask about the date they answered the questionnaire, which we today consider a limitation since it may have affected the answers. Lastly, the well‐known limitations of self‐report questionnaires with a possible bias from rating one's own behaviour should be acknowledged (Toomingas, Alfredsson, & Kilbom, 1997).

## 5 CONCLUSION

This study indicates that surgical patients who present with psychological distress during hospitalization are likely to show these symptoms up to 6 months postdischarge.

Development of appropriate interventions and research on those are needed. A prerequisite, as in all nursing interventions, is however a thorough psychological assessment before surgery.

## 6 RELEVANCE TO CLINICAL PRACTICE

The findings of this study have important clinical implications. First and foremost, patients presenting with symptoms of anxiety or depression at the hospital around discharge are in a vulnerable position and should receive follow‐up. It seems that postoperative symptoms and sleep have a pivotal influence on anxiety and depression in the recovery period. Therefore, a routine screening at hospital discharge and appropriate education should be emphasized. HADS is a good screening tool that is used in a variety of settings. During the follow‐up for patients presenting with symptoms of anxiety or depression, these should be reviewed and discussed with the patients. There is a need to further develop and study the effects of suitable interventions, and a thorough assessment before surgery, in this case psychological assessment should be done in order to detect patients at risk for postoperative psychological disturbances. The benefits might be decreased suffering, less overall medical cost and diminished chronic disability.

## CONFLICT OF INTEREST

The authors have declared no conflicts of interest.

## AUTHOR CONTRIBUTIONS

HS, SZ, BI, KB: Study design. HS, KB: Data collection. HS: Data analysis. HS, SZ, BI, KB: Manuscript preparation.

## Data Availability

Due to privacy and ethical concerns, neither the data nor the source of the data can be made available.
